# Reliability of the quality of life-aged care consumers (QOL-ACC) and EQ-5D-5L among older people using aged care services at home

**DOI:** 10.1186/s12955-024-02257-8

**Published:** 2024-05-30

**Authors:** Jyoti Khadka, Rachel Milte, Claire Hutchinson, Jenny Cleland, Julie Ratcliffe

**Affiliations:** 1 Health and Social Care Economics Group, Caring Futures Institute, College of Nursing and Health Sciences, Flinders University, Sturt North, GPO Box 2100, Adelaide, 5001 South Australia; 2 Registry of Senior Australians, South Australian Health and Medical Research Institute, Adelaide, South Australia

**Keywords:** Aged care, Quality of life, Quality of Care, QOL-ACC, Test–retest, Reliability, Standard error of measurement

## Abstract

**Purpose:**

The Quality of Life-Aged Care Consumers (QOL-ACC), a valid preference-based instrument, has been rolled out in Australia as part of the National Quality Indicator (QI) program since April 2023 to monitor and benchmark the quality of life of aged care recipients. As the QOL-ACC is being used to collect quality of life data longitudinally as one of the key aged care QI indicators, it is imperative to establish the reliability of the QOL-ACC in aged care settings. Therefore, we aimed to assess the reliability of the QOL-ACC and compare its performance with the EQ-5D-5L.

**Methods:**

Home care recipients completed a survey including the QOL-ACC, EQ-5D-5L and two global items for health and quality of life at baseline (T1) and 2 weeks later (T2). Using T1 and T2 data, the Gwet’s AC2 and intra-class correlation coefficient (ICC) were estimated for the dimension levels and overall scores agreements respectively. The standard error of measurement (SEM) and the smallest detectable change (SDC) were also calculated. Sensitivity analyses were conducted for respondents who did not change their response to global item of quality of life and health between T1 and T2.

**Results:**

Of the 83 respondents who completed T1 and T2 surveys, 78 respondents (mean ± SD age, 73.6 ± 5.3 years; 56.4% females) reported either no or one level change in their health and/or quality of life between T1 and T2. Gwet’s AC2 ranged from 0.46 to 0.63 for the QOL-ACC dimensions which were comparable to the EQ-5D-5L dimensions (Gwet’s AC2 ranged from 0.52 to 0.77). The ICC for the QOL-ACC (0.85; 95% CI, 0.77–0.90) was comparable to the EQ-5D-5L (0.83; 95% CI, 0.74–0.88). The SEM for the QOL-ACC (0.08) was slightly smaller than for the EQ-5D-5L (0.11). The SDC for the QOL-ACC and the EQ-5D-5L for individual subjects were 0.22 and 0.30 respectively. Sensitivity analyses stratified by quality of life and health status confirmed the base case results.

**Conclusions:**

The QOL-ACC demonstrated a good test-retest reliability similar to the EQ-5D-5L, supporting its repeated use in aged care settings. Further studies will provide evidence of responsiveness of the QOL-ACC to aged care-specific interventions in aged care settings.

**Supplementary Information:**

The online version contains supplementary material available at 10.1186/s12955-024-02257-8.

## Introduction

In 2021-22, approximately 1.3 million older Australians (aged 65 years and over) received aged care services either at home or in residential aged care facilities [1, 2]. Aged care in Australia is subsidised by the Commonwealth government with Aus$ 24.8 billion allocated to finance the aged care system in 2021-22 alone [2]. However, the Australian Aged Care system has been marred with numerous reports of abuse, neglect, poor service quality and sub-standard service delivery. In response to these concerns, the Australian Government established a Royal Commission investigation into Aged Care Quality and Safety in 2018 [3]. The Royal Commission conducted a 3 -year investigation and produced a damning final report in February 2021, concluding that the Australian Aged Care system was rife with sub-standard service delivery, poor quality services, inadequate monitoring and reporting and lacked public accountability [4]. The Royal Commission made a raft of recommendations to fundamentally reform the aged care system including recommendations to expand the existing quality indicators for on-going monitoring and public reporting of quality and safety in aged care [4]. 

Following the Royal Commission recommendations, the Australian Department of Health and Aged Care has expanded the existing quality indicators from five to eleven key indicators including two person-centred measures (quality of life and consumer experience) incorporated for the first time into the newly expanded National Aged Care Mandatory Quality Indicator Program (QI Program) [5]. The instruments that have been selected for the QI Program are the Quality of Life-Aged Care Consumers (QOL-ACC) and the Quality of Care-Aged Care Consumers (QCE-ACC). Participation in the QI Program is mandatory for all government subsidised residential aged care (nursing homes) service providers. Currently, a new set of QI indicators for home-based aged care services is also being trialed and both the QOL-ACC and QCE-ACC have been included in the feasibility study [6, 7].

Along with the QCE-ACC, the QOL-ACC was developed by our team using a ‘from the ground up’ approach by engaging with older people accessing aged care services in both home and residential care settings [8–10]. The QOL-ACC captures salient quality of life outcomes that matter most to older people and, which can also be improved through the care and support provided by aged care organisations [8, 11]. We have developed an older person specific preference-based scoring algorithm for the QOL-ACC, facilitating its application in economic evaluation to inform new and innovative cost-effective interventions that ensure high-quality care [12]. 

Ongoing evidence of the validity, reliability and responsiveness of the QOL-ACC instrument in a variety of aged care settings is important, given that the QOL-ACC is being operationalized nationally as a key QI indicator. The QOL-ACC has already demonstrated strong feasibility, internal consistency and construct validity both in home and residential aged care settings [10, 13, 14]. In addition to evidence of its validity, it is imperative to demonstrate that the QOL-ACC is a reliable instrument because it will be used to collect data longitudinally as a key aged care QI indicator.

An important reliability assessment is test-retest reliability [15]. For this, an instrument needs to be administered to the same sample twice within an appropriate time interval, with 2 weeks often considered as the optimal time interval [15, 16]. The underlying assumptions underpinning the test-rest reliability are (1) the two administrations should be independent from each other and (2) the gap between the two administrations should be such that it is unlikely for the respondents to experience any significant changes in their health and/or quality of life status but sufficiently long enough that respondents are not able to recall their first responses (i.e. a sufficient gap between two administrations to adjust for the potential for recall bias) [16, 17].

Reliability of the QOL-ACC has not been reported yet, but it is an important prerequisite psychometric property to show that the instrument is appropriate for use in repeated measurements longitudinally. To fill this gap in current knowledge, this study aimed to conduct comprehensive reliability assessments for the QOL-ACC including test-retest reliability and also used the same data to estimate standard error of measurement, smallest detectable change and test-retest agreement. In doing so, we sought to compare the QOL-ACC’s performance with the EuroQOL five dimensional five-levels (EQ-5D-5L, a widely used generic health related quality of life instrument) [18] to benchmark its reliability performance in older people accessing aged care services at home.

## Materials and methods

### Study population

The study population was older people receiving aged care at home either via the Commonwealth Home Support Programme (CHSP) or Home Care Package (HCP) Program. The CHSP provides entry-level aged care and support services such as meals and food preparation, household chores, personal care etc [19]. . HCPs offer tailored care services to older people with complex needs, and has four levels (HCP1 for basic care needs to HCP4 for high care needs) [2]. Both types of home-based care services are designed to support older Australians to live independently and safely at home for as long as possible.

An online panel company was used to recruit potential survey respondents. Older people receiving aged care services at home, nationally representative of older people in the community by gender and state/ territory of residence. Respondents were aged ≥ 65years, able to read and respond in English and living in Australia. The initial survey (test survey, T1) was self-completed by a total of 806 respondents. Two respondents who completed the survey too quickly (the survey completion time < 5 min) were excluded, hence data from 804 valid responses was used to develop an older person and aged care-specific preference-based value sets for the QOL-ACC [12]. Details of the first (test, T1) survey is already described elsewhere [12]. Of the 804 respondents, 83 (10.3%) self-completed the survey (re-test) approximately two weeks (ranged from 13 days to 16 days) following their completion of the initial survey. An approximate two-week time gap was chosen as optimal in older people accessing aged care services to balance between recall bias and control for any possibilities of significant decline in the respondents’ health and quality of life that might influence their responses.

### The test and retest surveys

Briefly, the test (first, T1) survey included a series of instruments (QOL-ACC, EQ-5D-5L, QCE-ACC), a discrete choice experiment facilitating the development of a preference based scoring algorithm (or value set) the QOL-ACC and a series of socio-demographic questions including age, gender, country of birth, living arrangement and self-report global items for general health and quality of life on the day of the survey administration rated on a 5-point scale (end points anchored as poor and excellent) [12]. Using postcode data (geographical areas of residence), two indices (Index of Relative Socio-economic Advantage and Disadvantage, IRSAD and Index of Education and Occupation, IEO) of socio-economic well-being were estimated using methodology described by the Australian Bureau of Statistics [20]. The retest (second, T2) survey included the QOL-ACC, EQ-5D-5L and two global items for general health and quality of life. We used the global items as anchor items to determine whether there was a significant shift in the self-reported health and quality of life between test and retest. Respondents who had 2 or more points difference in their responses to the global items for health or quality of life between test and retest surveys were excluded from the base case analysis. A unique identifier was used to link test and retest data. For the respondents who did and did not respond to the retest (T2) survey, there was no statistical difference in average age, frequency distribution of gender, country of birth, language spoken at home, types of home-based aged services used, living arrangement, self-rated health, or quality of life (Supplementary material Table 1). All respondents provided online consent prior to completing both the surveys.

### The instruments

#### QOL-ACC

The development, validation and valuation of the QOL-ACC as a new aged-care specific preference-based quality of life instrument have been previously described [8, 10, 12–14] Briefly, a mixed method approach using a traffic light system was used to integrate both qualitative (face validity) and quantitative (psychometric assessments) data to develop the final descriptive system for the QOL-ACC [8, 10, 11, 21] The QOL-ACC has 6 dimensions (mobility, pain management, independence, emotional well-being, social connections and activities) and rated on a 5 a five-point frequency scale (all of the time to none of the time). Application of DCE methodology with a large sample of older people receiving aged care services resulted in, a value set (range: -0.56 to 1.00) for the calculation of utilities for all QOL-ACC states [12], with a higher score representing a better quality of life.

#### EQ-5D-5L

The EQ-5D-5L is a widely used generic preference-based health-related quality of life utility instrument which has demonstrated superior feasibility and psychometric properties in populations of older people [22, 23]. It has five dimensions (mobility, self-care, usual activities, pain/discomfort and anxiety/depression) rated on a 5-point severity scale (no problems to extreme problems) [18]. For this study, we used the Australian pilot study preference weights developed by Norman et al. ) ranging from − 0.68 to 1.00) [24]. The EQ-5D-5L was administered alongside a visual analogue scale (VAS), the EQ VAS, a measure of self-reported health which ranges from 0 (worst possible health one can imagine) to 100 (best possible health one can imagine).

### Test-retest reliability

Test-retest reliability is a measure of temporal consistency of an instrument when the instrument is administered to the same respondents at two different time points. Test-retest analysis relies on the assumption that there is neither a memory effect nor true changes in the status of the respondents that may influence their responses over the repeated measurements [15]. Test–retest reliability of the QOL-ACC and EQ-5D-5L dimensions was examined by Gwet’s Agreement Coefficient (Gwet’s AC2 ) [25]. The extent to which the respective instruments produced the same overall utility scores during repeated administrations was measured by the Intraclass Correlation Coefficient (ICC) [26]. Besides Gwet’s AC2 and ICC, we also estimated standard error of measurement (SEM), smallest detectable change (SDC) and level of agreement between test and retest for the QOL-ACC and EQ-5D-5L [27]. 

### Standard error of measurement (SEM) and smallest detectable change (SDC)

SEM is defined as a random error in an instrument’s score that is not attributed to a true change in the measurement. The SEM provides a measure of variability within the framework for the test-retest assumptions; hence it can be used as an indicator for reliability. Like the standard deviation, the SEM can be interpreted as the observed value within which the theoretical “true” value lies. The interval between ± 1 SEM, ± 2 SEM and ± 3 SEM provide a probability of 68%, 95% and 99% of containing the true value respectively [28]. We used SEM to estimate the SDC for the QOL-ACC and EQ-5D. The SDC in essence can be defined as the magnitude of change in an instrument’s scores on repeated measures that needs to be observed to be confident that an observed change is real and not due to the measurement error or random variation.

### Bland-Altman plots

Bland-Altman plots were used to examine the test-retest agreement for the QOL-ACC and EQ-5D-5L (dimensional component and EQ VAS) separately. The plots provide a visual representation of the presence of any systematic difference between test and retest data for each instrument. The Y axis of the Bland-Altman plot represents the difference between test and retest while the X axis represents the mean of the test-retest scores. The limits of agreement (LOA) were calculated using the mean and the standard deviation of the differences between the test and retest: the limit of agreement = mean difference ± (standard deviation of the difference × 1.96) [29]. 

### Sample size estimation

To achieve an acceptable ICC of 0.80 with a confidence interval between 0.70 and 0.90, a sample size of 50 with a complete test-retest data is recommended [30–32]. To account for any attrition, missingness and to exclude individuals whose quality of life and health status might change between test and retest surveys, we targeted a complete test-retest survey data from a sample of *N* ≈ 80. Re-test data collection ceased when the target sample size was achieved.

### Statistical analysis

The analyses were carried out using STATA/SE, version 15.1. (Stata Corp LLC, Texas, USA). Socio-demographic characteristics were presented as percentage for categorical variables and with mean (standard deviation) or median (interquartile range) for continuous variables. To control for any influence due to change in health and quality of life on the test-retest results, we excluded respondents who changed their ratings by 2 or more levels on either of the global items for health and quality of life between the test and retest assessments.

Test–retest reliability of the QOL-ACC and EQ-5D-5L dimensions was examined by Gwet’s Agreement Coefficient (Gwet’s AC). We used Gwet’s AC2 because it is better at reflecting agreement for skewed ordinal data (e.g. very high or low prevalence of end category “no problems”) than Cohen’s kappa [33]. We interpreted Gwet’s AC2 as: < 0.00 poor, 0.00 to 0.20 slight, 0.21 to 0.40 fair, 0.41 to 0.60 moderate, 0.61 to 0.80 substantial and > 0.80 almost perfect agreement [33]. Test-retest reliability for the overall index score was assessed by calculating the ICC (95% confidence interval) using two-way random effects model (absolute agreement specified) [34]. An ICC of > 0.9, > 0.75 to 0.90, 0.5 to 0.75 and < 0.5 are considered as excellent, good, moderate and poor reliability respectively [34, 35].

The SEM was estimated by dividing standard deviation of the difference (SD_difference_) between test and retest scores by the square root of 2 [SEM = SD_difference_/√2]. For this study, the SDC was estimated for both for individual (SDC_ind_) and group level (SDC_group_). The SDC_ind_ was estimated using the formulae [SDC_ind_=1.96*√2*SEM]. The SDC_group_ was estimated by dividing the SDC_ind_ by the square root of the sample size [SDC_group_= SDC_ind_/√N] [28, 36]. Bland and Altman analyses were carried out to estimate mean differences and limits of agreement (LoA) for the QOL-ACC, EQ-5D-5L and EQ-VAS [29]. 

Sensitivity analyses were also performed to investigate whether any changes in self-reported health and quality of life between test and retest affected the main findings. In the sensitivity analyses, the respondents who changed their quality of life and health ratings by one level on global items of quality of life and health between test and retest were excluded and we ran separate analyses (1) respondents with no change in quality of life and health ratings (2) respondents with ≥ 1 level change in quality of life and health ratings. Additional sensitivity analyses were presented for the EQ-5D-5L with the latest Australian value set [37] and the US EQ-VT based value set that followed the EuroQoL valuation protocol [38]. Results were considered statistically significant where *p* ≤ 0.05.

## Results

Of the 83 respondents, five respondents who changed their ratings by two or more levels on the global items for health and quality of life between the test and retest surveys were excluded from the base case analysis. A total of 78 respondents were included, 56.4% (*n* = 44) were female, 56.4% (*n* = 44) were aged between 65 and 74 years, 76.9% (*n* = 60) were born in Australia, 25.6% used CHSP (*n* = 20), 44.9% (*n* = 35) were living alone and 60.3% (*n* = 47) made at least a small co-contribution to access home care services (Table 1).

**Table 1:** Characteristics of the respondents

Variables	*N* = 78 (100%)	
	Test (Time 1)	Re-test (Time 2)
**Gender, ** ***N*** **(%)**		
Male	34 (43.6)	
Female	44 (56.4)	
**Age, N (%)**		
65–74	44 (56.4)	
75–84	31 (39.7)	
85+	3 (3.8)	
Mean Age (SD)	73.6 (5.3)	
Median Age (IQR)	74 (70–77)	
**Country of birth, N (%)**		
Australia	60 (76.9)	
Outside Australia	18 (23.1)	
**Language spoken at home, N(%)**		
English	76 (97.4)	
Other than English	2 (2.6)	
**Care Packages and Levels, N (%)**		
Commonwealth Home Support Programme (CHSP)	20 (25.6)	
Home Care Package- Level 1	13 (16.7)	
Home Care Package- Level 2	9 (11.5)	
Home Care Package- Level 3	5 (6.4)	
Home Care Package Level 4	1 (1.3)	
Unsure	30 (38.5)	
**Living arrangements, N (%)**		
Living alone	35 (44.9)	
Living with spouse/partner	40 (51.3)	
Living with others (not relatives)	3 (3.8)	
**Informal carer availability**		
Yes	25 (32.1)	
No	53 (67.9)	
**Highest educational qualification, N (%)**		
No qualifications	5 (6.4)	
Completed high school	34 (43.6)	
Undergraduate degree/Professional qualification	23 (29.5)	
Postgraduate qualification	12 (15.4)	
*Other	4 (5.1)	
**Hours of support services per week, N(%)**		
≤ 2 h	57 (73.1)	
3–4 h	8 (10.3)	
5–9 h	7 (9.0)	
≥ 10 h	6 (7.7)	
**Co-contribution for the care they receive, N(%)**		
None	26 (33.3)	
Make a small contribution	47 (60.3)	
Pay for all of my care	5 (6.4)	
Types of services being received**, N(%)		
Meals or help with cooking	8 (10.2)	
Cleaning	60 (76.9)	
Shopping	7 (9.0)	
Transportation	13 (16.7)	
Gardening	31 (39.7)	
Personal care	3 (3.8)	
Home nursing	1 (1.3)	
Group social activities	6 (7.7)	
*Others	10 (11.5)	
**SEIFA-IRSEAD quintiles, N(%)**		
1 (least advantaged)	15 (19.2)	
2	15 (19.2)	
3	18 (23.1)	
4	20 (25.6)	
5 (most advantaged)	10 (12.8)	
**SEIFA- IEO quintiles, N(%)**		
1 (least advantaged)	17 (21.8)	
2	12 (15.4)	
3	21 (26.9)	
4	17 (21.8)	
5 (most disadvantaged)	6 (7.7)	
**Self-reported health, N (%)**		
Excellent	2 (2.6)	4 (5.1)
Very good	18 (23.1)	16 (20.5)
Good	29 (37.2)	26 (33.3)
Fair	22 (28.2)	22 (28.2)
Poor	7 (9.0)	10 (12.8)
**Self-reported quality of life, N(%)**		
Excellent	4 (5.1)	5 (6.4)
Very good	29 (37.2)	25(32.1)
Good	23 (29.5)	27 (34.6)
Fair	19 (24.4)	19 (24.4)
Poor	3 (3.8)	2 (2.6)

Note: *SEIFA-IRSEAD: Social Economic Indices for Areas- Index of Relative Socio-Economic Advantage and Disadvantage; SEIFA-IEO: Social Economic Indices for Areas- Index for Education and Occupation.*

QOL-ACC: Quality of Life-Aged Care Consumers. ** individual might be receiving more than one service types and the percentage for a specific service type was estimated out of *N* = 78.

*others refer to option not listed in the survey

### Test-retest reliability

The Gwet’s AC2 for the QOL-ACC and EQ-5D-5L’s dimensions ranged from 0.46 to 0.63 and 0.52 to 0.77 respectively (Table 2). Two of the QOL-ACC’s dimensions (mobility and social connections) and three of the EQ-5D-5L dimensions (mobility, self-care and anxiety/depression) demonstrated a substantial agreement whereas all other dimensions reported a moderate agreement. Both the QOL-ACC (ICC = 0.85, 95% CI = 0.77–0.90) and EQ-5D-5L (ICC = 0.83, 95% CI = 0.74–0.88) index values demonstrated good test-retest reliability whereas the EQ VAS (ICC = 0.70, 95% CI = 0.56–0.80) showed moderate reliability (Table 3).

**Table 2:** Gwet’s AC2 of the of the QOL-ACC and EQ-5D-5L dimensions (*N* = 78)

QOL-ACC dimensions (*N* = 78)	Gwet’s AC2 (95% CI)	EQ-5D-5L dimensions	Gwet’s AC2 (95% CI)
Mobility	0.63 (0.50–0.76)	Mobility	0.63 (0.50–0.75)
Pain management	0.56 (0.43–0.70)	Self-care	0.77 (0.66–0.88)
Emotional	0.57 (0.43–0.70)	Usual activities	0.54 (0.40–0.67)
Independence	0.46 (0.32–0.60)	Pain/ discomfort	0.52 (0.39–0.66)
Social connections	0.63 (0.50–0.76)	Anxiety/depression	0.61 (0.47–0.74)
Activities/Hobbies	0.48 (0.34–0.62)		

Note: QOL-ACC = Quality of Life- Aged Care Consumers; EQ-5D-5L = EuroQoL-5 dimensions 5 levels

**Table 3:** Standard error of measurement, smallest detectable change and intraclass correlation coefficient of the QOL-ACC, EQ-5D-5L and EQ VAS. (*N* = 78)

Instrument	T1, mean (95% CI)	T2, mean (95% CI)	ICC (95% CI)^#^	Mean difference (95% CI)	SD_diff_	SEM	SDC_Ind_	SDC_group_	95% LOA
QOL-ACC	0.72 (0.66 to 0.77)	0.76 (0.71 to 0.80)	0.85 (0.77 to 0.90)	0.03 (-0.01 to 0.06)	± 0.12	0.08	0.22	0.02	-0.20 to 0.28
EQ-5D-5L	0.62 (0.56 to 0.68)	0.63 (0.57 to 0.69)	0.83 (0.74–0.88)	0.01 (-0.02 to 0.05)	± 0.16	0.11	0.30	0.03	-0.30 to 0.32
EQ VAS	60.9 (57.3 to 65.0)	63.2 (55.9 to 67.5)	0.70 (0.56 to 0.80)	1.19 (-3.08 to 5.47)	± 19.0	13.4	37.2	4.21	-36.0 to 38.4

QOL-ACC = Quality of Life: Aged Care Consumers; EQ-5D-5L = EuroQOL 5 Dimension 5Level; EQ VAS = EuroQOL Visual Analogue Scale; SD_diff_= standard deviation of the difference; SEM = standard error of measurement; SDC = smallest detectable change; ICC = Intraclass correlation coefficient; LOA = Limit of Agreement; T1 = Time 1, T2 = Time 2

### The SEM and SDC

The SEM for the QOL-ACC utility scores was 0.08, meaning that there is a 68% confidence (± 1 SEM) that the true utility value for an individual was within ± 0.08, and 95% confidence (± 2 SEM) that true utility value for an individual was within ± 0.16. For the EQ-5D-5L and the VAS, the SEM were ± 0.11 and ± 13.4 respectively (Table 3).

The SDC_ind_ and SDC_group_ for the QOL-ACC were 0.22 and 0.02 respectively. These values mean that the utility score of an individual and the complete sample would have to change by more than 0.22 and 0.02 respectively before an observed change may be considered as a true change beyond the measurement error. The SDC_ind_ and SDC_group_ for the EQ-5D-5L were 0.30 and 0.03 respectively. For the EQ VAS, the SDC_ind_ and SDC_group_ were 37.2 and 4.21 respectively (Table 3).

### Bland and Altman analysis

**Fig. 1:**
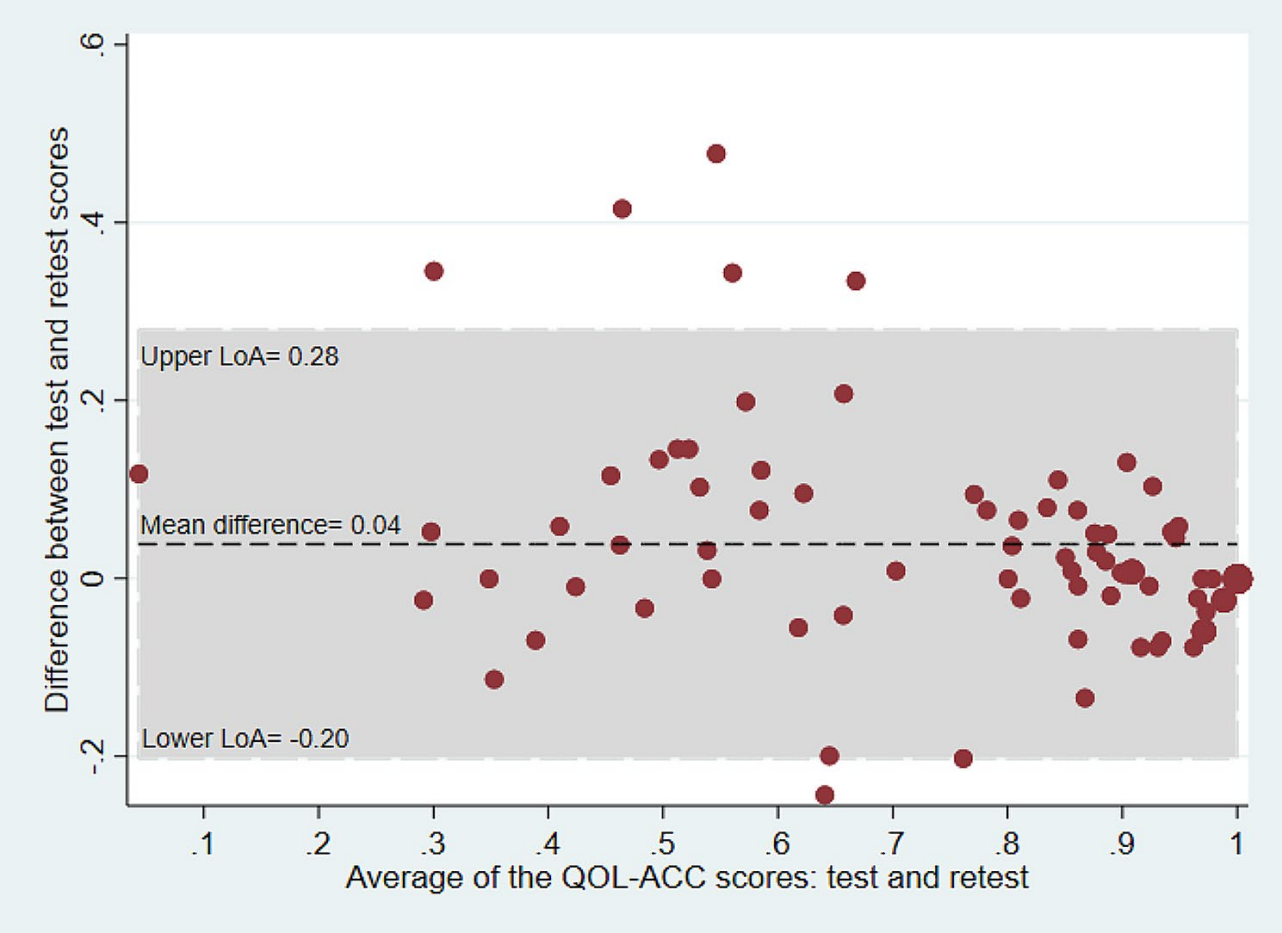
Bland and Altman plot for the QOL-ACC, average of the QOL-ACC index scores between test and re-test plotted against the difference in scores. LoA = Limits of agreement

**Fig. 2:**
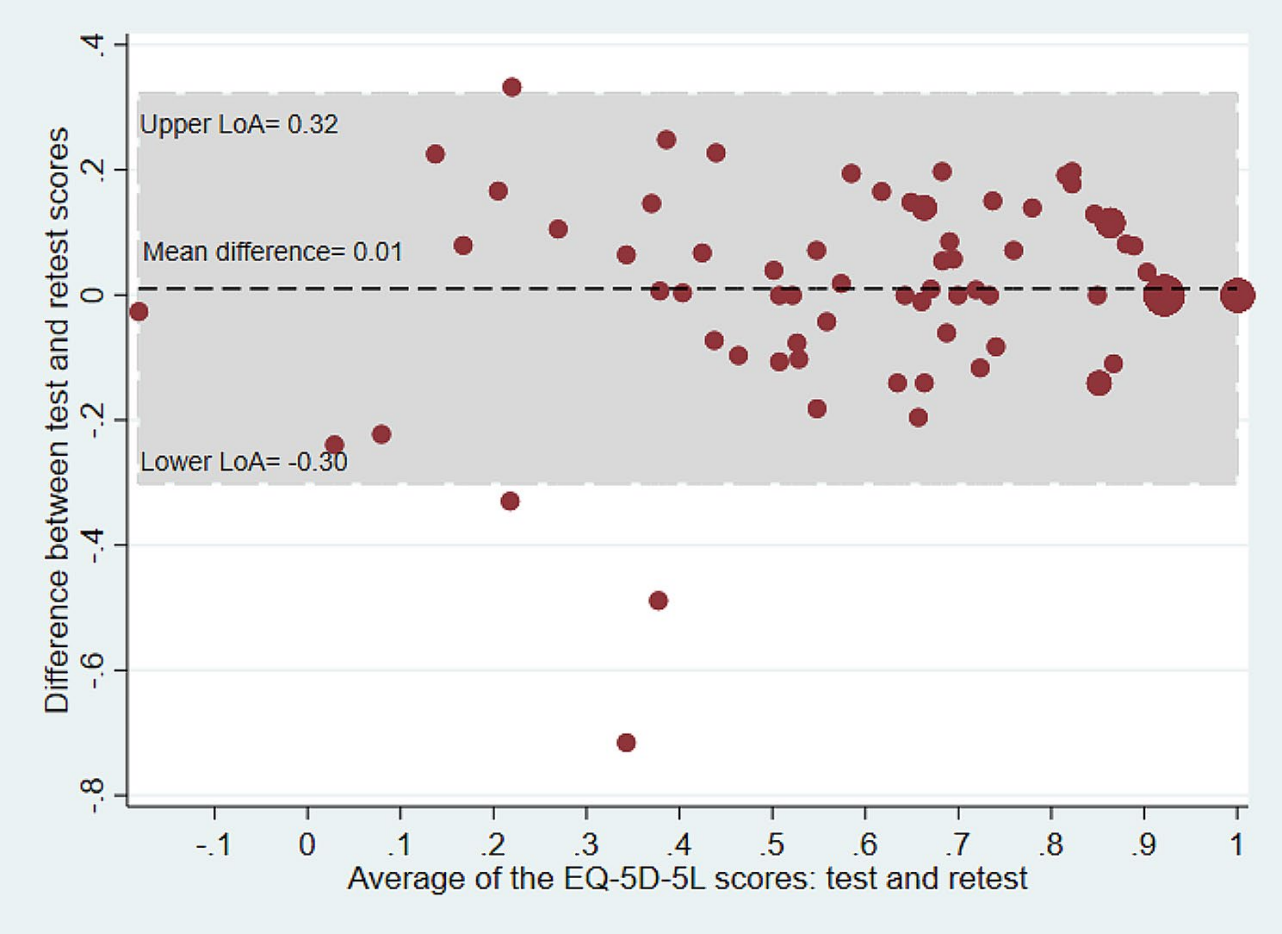
Bland and Altman plot for the EQ-5D-5L, average of the EQ-5D-5L index scores between test and re-test plotted against the difference in scores. LoA = Limits of agreement

**Fig. 3:**
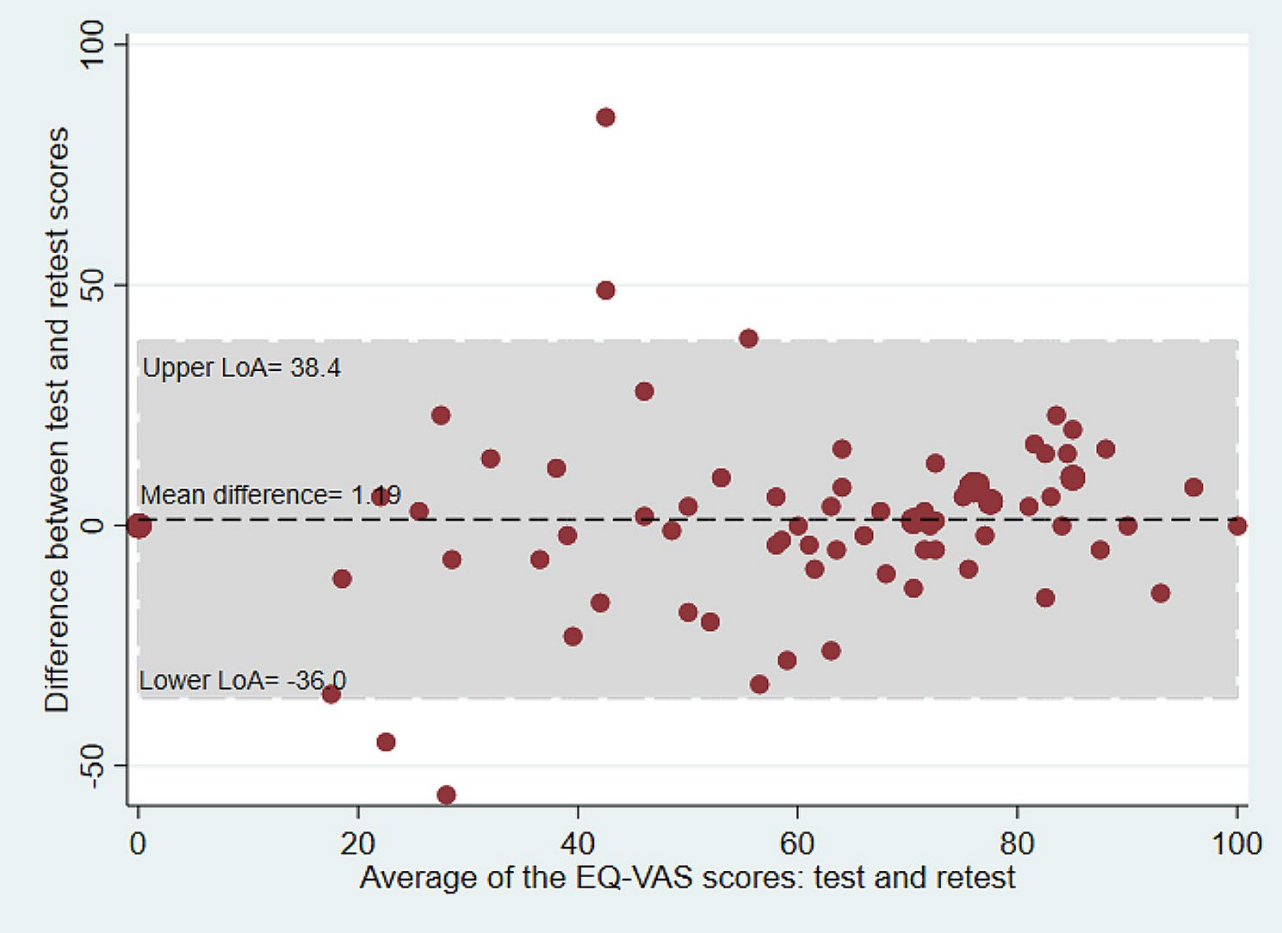
Bland and Altman plot for the EQ-VAS, average of the EQ-VA index scores between test and re-test plotted against the difference in scores. LoA = Limits of agreement

The mean difference between test and retest survey for the QOL-ACC was 0.03 (95% CI=-0.01 to 0.06) and the 95% LoA agreement was between − 0.20 and 0.28 (Table 3; Fig. 1). The mean difference for the EQ-5D-5L was 0.01 (95% CI =-0.02 to 0.05) and the 95% LoA was between − 0.30 and 0.32 (Table 3; Fig. 2). Similarly, the mean difference for the EQ-VAS was 1.19 (95% CI= -3.08 to 5.47) and the 95% LoA was between − 36.0 and 38.4 (Table 2; Fig. 3). The LOA spanned zero for both the QOL-ACC, EQ-5D-5L and EQ-VAS, indicating nosystematic biases between the test and retest administrations.

### Sensitivity analysis

Of the 78 respondents, *N* = 48 did not change their quality of life ratings and *N* = 56 did not change their health ratings between the test and retest administrations for the global item of quality of life and health respectively. Separate sensitivity analyses were conducted to assess Gwet’s AC2 and ICC of scores reported at both assessment points between respondents who reported a change and respondents who did not report a change in quality of life and health. The results demonstrated that test-retest reliability statistics at dimension level (Gwet’s AC2, Supplementary Table 2, Table 3) and the overall scores (ICC, Supplementary Table 4) for both the QOL-ACC and the EQ-5D-5L were similar to the base case results (*N* = 78). Additional sensitivity analyses that estimated ICC values for the EQ-5D-5L with the new Australian value set and the US VT-based value set (Supplementary Table 5 were also similar results to the base case results.

## Discussion

Further to the empirical evidence of strong content validity [10, 11] and psychometric performance of the QOL-ACC in aged care settings, [8, 13, 14] this study demonstrated that the QOL-ACC is also a reliable instrument, supporting its repeated and longitudinal application to assess quality of life for older people in home and community based aged care settings. The reliability statistics for the QOL-ACC were either similar or comparable to the EQ-5D-5L, indicating that the QOL-ACC performed as good as the EQ-5D-5L in our study population.

The overall index score of the QOL-ACC exhibited a very high test-retest reliability with an ICC value of 0.85 with its lower bound of the 95% CI exceeding 0.75 which is the cut off value for high reliability. Such a high degree of confidence in the test-retest reliability for the QOL-ACC is encouraging when compared with other preference-based instruments [39, 40]. For example, a study by van Leeuwen et al. reported lower test-retest ICC values (< 0.80) for the three preference-based instruments (EQ-5D-3L, ASCOT and ICECAP-O) in a test-retest study conducted in older frail people living in home. Among the three instruments, the ASCOT had an ICC agreement value of 0.71 but its lower bound of the 95% confidence interval was significantly lower than the acceptable 0.70 (i.e., 0.60) [39]. In another study, the ICECAP-O had an ICC of 0.80 but its lower bound of 95% CI for the ICC was below 0.70 (i.e. 0.62) [40]. Further, the ICC agreement for the QOL-ACC was higher than the EQ-5D-5L in the current study suggesting that the QOL-ACC is a highly reliable instrument in Australian aged care settings. Interestingly, the value of the EQ-5D-5L index values in the current study is similar to that reported in patient populations with care needs but much higher than reported in general population [41–43]. Given that our study population were aged care recipients who were also likely to have co-morbidities, our study findings are comparable to studies that have used EQ-5D-5L in populations of older people with health conditions [41–43]. 

Given that the QOL-ACC demonstrated smaller SEM and SDC values in this study relative to the EQ-5D-5L and the EQ VAS (Table 3), it is likely that a relatively small change in its index score can be considered as a true change in scores rather than a change due to measurement error under the assumptions adopted for test-retest (i.e. there was no significant change in the health and/or quality of life of respondents between the two measurement time points). The SEM of the EQ-5D-5L was slightly larger than the QOL-ACC meaning that a larger sample size would be required to detect changes than with the QOL-ACC. We reported the SDC both at individual and group levels, however for cost effectiveness analysis, changes at a group level are more relevant [31]. The knowledge of SDC is important to interpret longitudinal data collected with the QOL-ACC, however this value does not imply that the change in QOL-ACC scores could be considered as a minimal important difference (MID) score, as important changes could be either smaller or larger than the SDC and tested on a different assumption that the study population has likely changed their quality of life after an intervention. Further longitudinal studies to assess the responsiveness of the QOL-ACC to detect changes in quality of life over time are needed to identify the MID. As expected, due to widely reported concerns with the validity and inconsistent test-retest reliability, [44, 45] it was unsurprising that the EQ-VAS demonstrated lower reliability, large SEM and SDC values in this study population.

The mean index scores of the QOL-ACC both at test and retest time points were much higher than that of the EQ-5D-5L. The difference in mean scores may be due to differences in the constructs that these two instruments assess: the QOL-ACC is an older person-specific quality of life instrument whereas the EQ-5D-5L is a generic health related quality of life instrument designed for application with adults of all ages. It is likely that the QOL-ACC was capturing aspects of quality of life associated with aged care that are not captured by the five dimensions of EQ-5D-5L.

Test-retest reliability should be assessed in a stable study population with an appropriate time interval between the two measurements. We assumed that two weeks was an optimal time interval for this study. However, it is possible that change in respondents’ health and quality of life status might have affected the test-retest estimates. To ensure the robustness of our findings, we carried out sensitivity analyses to assess potential changes in health and quality of life status by excluding respondents who changed their self-reported quality of life and health ratings even by a single point between test and retest surveys. In these sub-samples, we did not find any significant differences in test-retest statistics (Supplementary Tables 2 and 3) providing additional confidence in our main findings. Further, Bland and Altman plots demonstrated that the mean difference between test and retest was close to zero for both the instruments, indicating that there was no systematic bias in the data. Interestingly, in the sensitivity analyses, individuals with no change in self-reported quality of life had higher ICC agreement values with the QOL-ACC than those with no change in self-reported health ratings. These findings were opposite for the EQ-5D-5L, that is, the respondents with no change in self-reported health had higher ICC agreement than those with no change in than quality of life and vice versa for the QOL-ACC (Supplementary Table 3). These findings may reinforce the fact that these instruments capture different concepts, that is, the EQ-5D-5L is a health-related quality of life instrument whereas the QOL-ACC is an older person and aged care specific instrument with more emphasis on psychosocial aspects of quality of life.

A major strength of the study was that it was adequately powered in terms of sample size when compared to other studies that reported test-retest analysis [46, 47] Our sample size was higher than that proposed in guidelines, a minimum of 50 respondents is considered adequate for assessing test-rest reliability [17]. There are several limitations to highlight. Our study sample was drawn from a pool of older people with access to internet and who were English speaking, therefore it is not completely representative of the population of older people receiving aged care services at home. The Australian Bureau of Statistics indicate that whilst most older Australians are regular internet users, a significant minority (38% in 2018) are not in the past three months. Furthermore, respondents self-completed the survey online and hence we were not able to verify whether they understood the survey well and provided accurate responses. Further, it is likely that the study findings may have been influenced by the order in which the instruments were administered. As the QOL-ACC was always administered first both at test and retest surveys, it was not possible to assess whether the order of administration had any significant impact on our results. Further research could address this issue through methods such as randomization of instrument and counterbalancing. Our group is currently undertaking a body of work to translate and validate the QOL-ACC into other non-English languages and to produce easy-read/pictorial versions of the instrument for older people with cognitive impairment and dementia.

In conclusion, this study has demonstrated that the QOL-ACC is a reliable instrument with good temporal consistency, supporting its repeated use as a key quality indicator among older people accessing aged care services at home. This study also supports the adoption of the QOL-ACC as an outcome measure in economic evaluation for aged care interventions where a broader aim of improving quality of life is the major focus. Further reliability assessment of the QOL-ACC in residential aged care settings is warranted. Also, future studies need to explore its responsiveness to provide evidence of its applicability for economic evaluation of aged care specific interventions in trials and cohort studies.

### Electronic supplementary material

Below is the link to the electronic supplementary material.

Supplementary Material 1

## Data Availability

No datasets were generated or analysed during the current study.
