# Supplementary material for: Reliability of the quality of life-aged care consumers (QOL-ACC) and EQ-5D-5L among older people using aged care services at home
Source: Health Qual Life Outcomes. 2024 May 30;22:40. doi: 10.1186/s12955-024-02257-8 (PMC11140901; doi:10.1186/s12955-024-02257-8)
Supplement: Supplementary file 1 — Supplementary Material 1 [file 12955_2024_2257_MOESM1_ESM.docx]

**Supplementary Table 1:** Comparing socio-demographic characteristics between those who did and did not complete the second (retest) survey

| **Variables** | **N=78 (100%)**  **Completed both Test and retest survey.** | **N=721 (100%)**  **Completed the first survey only** | **P values** |
| --- | --- | --- | --- |
| **Gender, N (%)** |  |  |  |
| Male | 34 (43.6) | 317 (44.0) | 0.94# |
| Female | 44 (56.4) | 404 (56.0) |  |
| **Age, N (%)** |  |  |  |
| 65-74 | 44 (56.4) | 381 (52.9) | 0.38# |
| 75-84 | 31 (39.7) | 284 (39.5) |  |
| 85+ | 3 (3.8) | 54 (7.5) |  |
| Mean Age (SD) | 73.6 (5.3) | 74.7 (6.1) | 0.11@ |
| Median Age (IQR) | 74 (70-77) | 74 (70-79) |  |
| **Country of birth, N (%)** |  |  |  |
| Australia | 60 (76.9) | 526 (72.9) | 0.57# |
| Outside Australia | 18 (23.1) | 195 (27.1) |  |
| **Language spoken at home, N(%)** |  |  |  |
| English | 76 (97.4) | 711 (98.7) | 0.39# |
| Other than English | 2 (2.6) | 9 (1.3) |  |
| ***Care Packages and Levels, N (%)** |  |  |  |
| Commonwealth Home Support Programme | 20 (25.6) | 188 (26.1) | 0.75# |
| Home Care Package- Level 1 | 13 (16.7) | 118 (16.4) |  |
| Home Care Package- Level 2 | 9 (11.5) | 83 (11.5) |  |
| Home Care Package- Level 3 | 5 (6.4) | 55 (7.6) |  |
| Home Care Package Level 4 | 1 (1.3) | 33 (4.6) |  |
| Unsure | 30 (38.5) | 244 (33.8) |  |
| **Living arrangements, N (%)** |  |  |  |
| Living alone | 35 (44.9) | 300 (41.6) | 0.60# |
| Living with spouse/partner | 40 (41.43) | 365 (50.6) |  |
| Living with relatives | - | 39 (4.8) |  |
| Living with others (not relatives) | 3 (3.8) | 19 (2.6) |  |
| **Self-reported health, N (%)** |  |  |  |
| Excellent | 2 (2.6) | 12 (1.7) | 0.39# |
| Very good | 18 (23.1) | 124 (17.2) |  |
| Good | 29 (37.2) | 241 (33.4) |  |
| Fair | 22 (28.2) | 268 (37.2) |  |
| Poor | 7 (9.0) | 76 (10.5) |  |
| **Self-reported quality of life, N(%)** |  |  |  |
| Excellent | 4 (4.8) | 43 (5.9) | 0.59# |
| Very good | 29 (37.2) | 207 (28.7) |  |
| Good | 23 (29.5) | 267 (37.0) |  |
| Fair | 19 (24.4) | 177 (24.6) |  |
| Poor | 3 (3.6) | 27 (3.7) |  |
| **Note:*** individual might be receiving more than one service types and the percentage for a specific service type was estimated out of the total of the respondents. #Pearson’s Chi2 test; @Wilcoxon rank-sum test | | | |

**Supplementary Table 2:** Gwet’s AC2 of the QOL-ACC and EQ-5D-5L dimensions for those who did not change self-reported quality of life (N=48) and health (N=56) ratings between test and retest administration

| **QOL-ACC dimensions** | **Gwet’s AC2 (95% CI)** | | **EQ-5D-5L dimensions** | **Gwet’s AC2 (95% CI)** | |
| --- | --- | --- | --- | --- | --- |
|  | **No change in QOL** | **No change in health** |  | **No change in QOL** | **No change in health** |
| Mobility | 0.61 (0.45-0.78) | 0.61 (0.46-0.77) | Mobility | 0.67 (0.51-0.83) | 0.59 (0.43-0.74) |
| Pain management | 0.54 (0.43-0.69) | 0.55 (0.39-0.71) | Self-care | 0.78 (0.64-0.92) | 0.85 (0.74-0.96) |
| Emotional | 0.47 (0.28-0.65) | 0.59 (0.43-0.75) | Usual activities | 0.52 (0.35-0.70) | 0.57 (0.41-0.73) |
| Independence | 0.51 (0.34-0.69) | 0.38 (0.22-0.54) | Pain/ discomfort | 0.61 (0.43-0.77) | 0.48 (0.32-0.64) |
| Social connections | 0.56 (0.36-0.75) | 0.54 (0.35-0.72) | Anxiety/depression | 0.68 (0.49-0.83) | 0.58 (0.41-0.74) |
| Activities/Hobbies | 0.51 (0.33-0.68) | 0.47 (0.31-0.64) |  | | |
| *QOL-ACC= Quality of Life: Aged Care Consumers; EQ-5D-5L= EuroQOL 5 Dimension 5Level; EQ VAS= EuroQOL Visual Analogue Scale, QOL= Quality of Life* | | | | | |

**Supplementary Table 3:** Gwet’s AC2 of the QOL-ACC and EQ-5D-5L dimensions for those who did change (by ≥1 level) self-reported quality of life (N=30) and health (N=22) ratings between test and retest administrations

| **QOL-ACC dimensions** | **Gwet’s AC2 (95% CI)** | | **EQ-5D-5L dimensions** | **Gwet’s AC2 (95% CI)** | |
| --- | --- | --- | --- | --- | --- |
|  | **≥1 level change in QOL** | **≥1 level change in health** |  | **≥1 level change in QOL** | **≥1 level change in health** |
| Mobility | 0.69 (0.49-0.89) | 0.74 (0.53-0.94) | Mobility | 0.57 (0.35-0.79) | 0.76 (0.56-0.97) |
| Pain management | 0.57 (0.37-0.78) | 0.57 (0.32-0.82) | Self-care | 0.77 (0.60-0.93) | 0.62 (0.39-0.86) |
| Emotional | 0.67 (0.47-0.87) | 0.47 (0.21-0.73) | Usual activities | 0.54 (0.33-0.75) | 0.43 (0.18-0.69) |
| Independence | 0.39 (0.16-0.62) | 0.68 (0.45-0.90) | Pain/ discomfort | 0.40 (0.18-0.61) | 0.58 (0.33-0.82) |
| Social connections | 0.67 (0.48-0.87) | 0.72 (0.52-0.94) | Anxiety/depression | 0.51 (0.29-0.74) | 0.67 (0.44-0.90) |
| Activities/Hobbies | 0.45 (0.24-0.67) | 0.51 (0.27-0.75) |  | | |
| *QOL-ACC= Quality of Life: Aged Care Consumers; EQ-5D-5L= EuroQOL 5 Dimension 5Level; EQ VAS= EuroQOL Visual Analogue Scale, QOL= Quality of Life* | | | | | |

**Supplementary Table 4:** The intraclass correlation coefficient (ICC) of the QOL-ACC, EQ-5D-5L and EQ VAS for those who did change (by ≥1 level) and did not change self-reported quality of life and heath ratings between test and retest administrations

| **Instrument** |  | **ICC (95% CI)** | | |  |
| --- | --- | --- | --- | --- | --- |
|  | **No change in QOL N=48** | | **≥1 level change in QOL, N=30** | **No change in health N=56** | **≥1 level change in health, N=22** |
| QOL-ACC | 0.89 (0.81-0.94) | | 0.78 (0.60-0.88) | 0.84 (0.72-0.91) | 0.77 (0.55 -0.88) |
| EQ-5D-5L | 0.85 (0.80-0.93) | | 0.78 (0.60-0.88) | 0.89 (0.82-0.93) | 0.71 (0.36 -0.87) |
| EQ VAS | 0.65 (0.45-0.79) | | 73 (0.52-0.85) | 0.70 (0.53-0.81) | 0.68 (0.41-0.84) |
| *QOL-ACC= Quality of Life: Aged Care Consumers; EQ-5D-5L= EuroQOL 5 Dimension 5Level; EQ VAS= EuroQOL Visual Analogue Scale, QOL= Quality of Life* | | | | | |

**Supplementary Table 5:** The intraclass correlation coefficient (ICC) of the EQ-5D-5L between test and retest administration

| **Instrument** | **ICC (95% CI) (N=78)** | |
| --- | --- | --- |
|  | **Australian new value set*** | **US VT-based value set**** |
| EQ-5D-5L | 0.81 (0.74-0.85) | 0.83 (0.75-0.88) |
| ***Note:*** **new Australian Value set by Norman et al (2023) (ref: Norman R, Mulhern B, Lancsar E, Lorgelly P, Ratcliffe J, Street D, et al. The Use of a Discrete Choice Experiment Including Both Duration and Dead for the Development of an EQ-5D-5L Value Set for Australia. Pharmacoeconomics. 2023;41(4):427-38.*  *US value set by Pickard et al 2019 (ref: Pickard AS, Law EH, Jiang R, Pullenayegum E, Shaw JW, Xie F, et al. United States Valuation of EQ-5D-5L Health States Using an International Protocol. Value Health. 2019;22(8):931-41.*  . | | |
